# Editorial: Solanaceae VIII: biodiversity, climate change and breeding

**DOI:** 10.3389/fgene.2023.1348372

**Published:** 2023-12-20

**Authors:** Péter Poczai, Nunzio D’Agostino, Rocio Deanna, Ezio Portis

**Affiliations:** ^1^ Botany and Mycology Unit, Finnish Museum of Natural History, University of Helsinki, Helsinki, Finland; ^2^ Museomics Research Group, Helsinki Institute of Life Science (HiLIFE), University of Helsinki, Helsinki, Finland; ^3^ University of Naples Federico II, Department of Agricultural Sciences, Portici, Italy; ^4^ University of Colorado, Boulder, CO, United States; ^5^ Instituto Multidisciplinario de Biología Vegetal (IMBIV, CONICET-UNC), Córdoba, Argentina; ^6^ Department of Agricultural, Forest and Food Sciences (DISAFA), Plant Genetics, University of Turin, Grugliasco, Italy

**Keywords:** *Capsicum*, mapping populations, near-isogenic lines (NILs), persistent and acute viruses, *Physalis*, quantitative trait loci (QTL), resistance hotspots, *Solanum*

**FIGURE 1:**
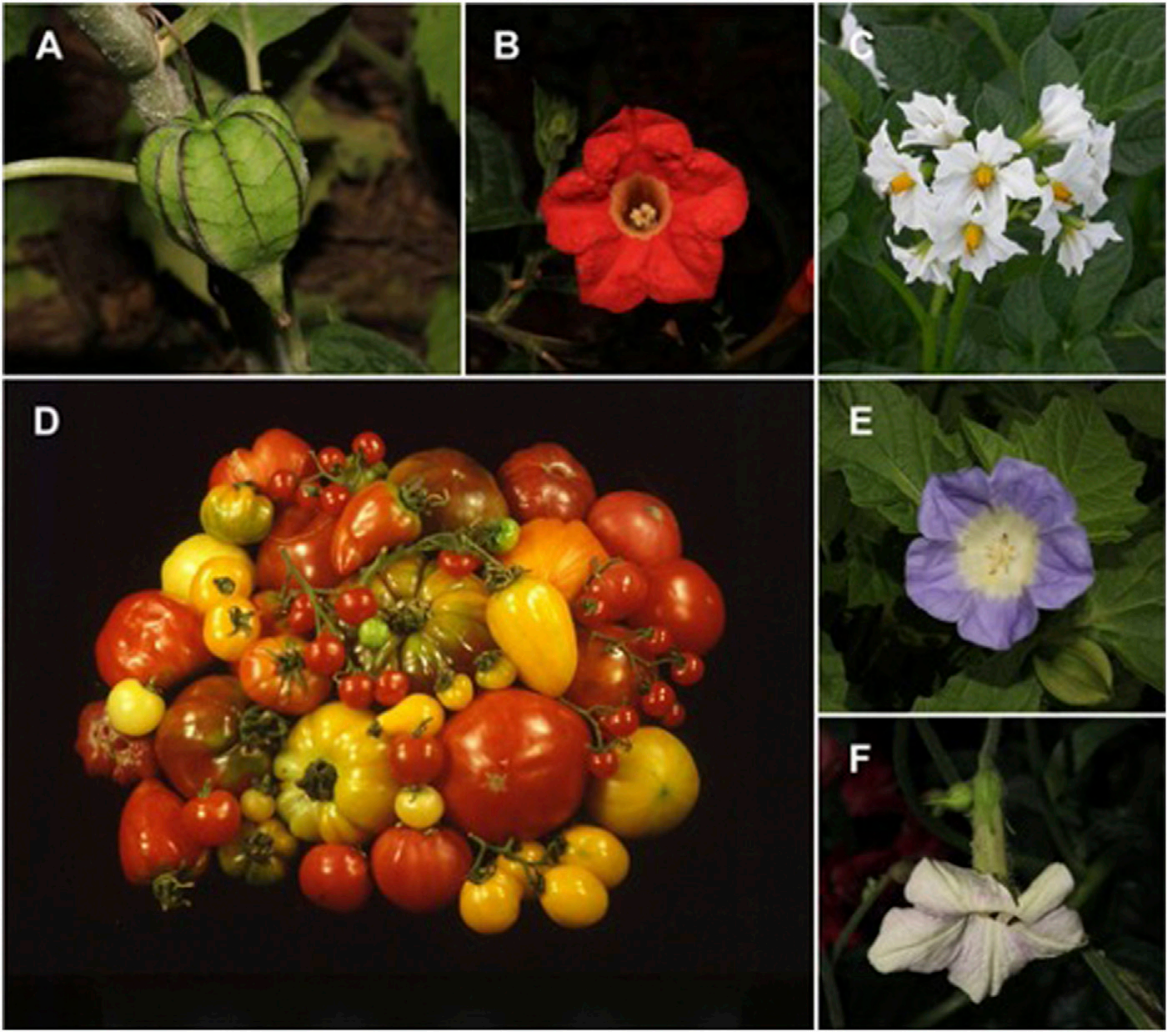
Diversity of Solanaceae crops and wild relatives. **(A)** *Physalis angulata* from Salta, Argentina. **(B)** *Plowmania nyctaginoides* from Chiapas, Mexico. **(C)** *Solanum tuberosum*, cultivated. **(D)** *Solanum lycopersicum*, cultivated. **(E)** *Nicandra physalodes*, cultivated, from the Chelsea Physic Garden. **(F)** *Nicotiana mutabilis*, cultivated, from the Chelsea Physic Garden. A-B, E-F photographs by R. Deanna; C and D courtesy of Jaime Prohens, Universitat Politècnica de València (UPV - Spain).

The Solanaceae is a widely recognized flowering plant family by its economically important crops and broad diversity (Figure 1). It encompasses many of the most useful plants—potato, tomato, eggplant and pepper—but it also features some of the most noxious—tobacco, henbane—while other species are beautiful ornamental species, like petunias, painted tongue flowers and chalice vines. Solanaceous plants play a vital part in human nutrition and global economies, but they also provide raw materials and resources for medicines and other purposes. The loss of this unique diversity has accelerated to an unprecedented extent due to climate change and human activity. According to the International Union for Conservation of Nature (IUCN), 7% of solanaceous species are critically endangered, 3% are near threatened or vulnerable, and seven species are already extinct in the wild (IUCN, 2023). Since the contributions of wild relatives of crops to food security depend on their conservation and accessibility for use, it is critical to safeguard their genetic diversity from extinction in the field.

Tackling the loss of this diversity to sustain ecosystem services needs technological advances accompanied by societal changes to better manage our natural capital before unexpected impacts start snowballing into destructive consequences (Dee et al., 2017). The remarkable rate of single nucleotide polymorphism identification is enhancing our capacity to understand phenotypic diversity at the genomic level. Genome-to-phenome research is crucial for detecting allelic variation for a wide range of traits and accelerating the adaptation of Solanaceae to climate change (Ichihashi and Sinha, 2014). Candidate genes and alleles can undergo functional characterization studies via “loss-of-function” and “gain-of-function” techniques (Berardi et al., 2021). Although there are no readily useable *in vitro* regeneration and transformation protocols for all Solanaceae (Cardi et al., 2017), genome editing technologies are increasingly being used to study gene function and accelerate gene transfer.

The densely interspersed chiasma that these paths produce in the transition from academic to applied sciences and *vice versa* highlight the tremendous potential of this plant family to bridge agendas across various research settings, as well as the professional prospects that this potential offers. This emphasizes an often overlooked yet still significant feature of the Solanaceae that has been highlighted in this Research Topic: the astonishing diversity with which this plant family is bringing together heterogeneous and idiosyncratic elements of technoscientific culture and knowledge in order to address biological phenomena from biodiversity to crop breeding under an alarmingly changing climate.

Recent studies by Carrizo García et al. and Hill et al. illuminate the historical footprints shaping *Capsicum* species’ diversification and the quest for mechanized harvesting-friendly green chili pepper varieties. Carrizo García et al. proposed a new model for understanding the spatio-temporal patterns of diversification for *Capsicum* species based on the analysis of single nucleotide polymorphisms derived from restriction-site associated DNA (RAD) genotyping. The analysis, which involved over 50 samples representing 36 species of *Capsicum* from all currently recognized clades of the genus, has provided an updated phylogenetic hypothesis of relationships within the genus *Capsicum*. Ancient expansion events in the Miocene are linked to the emergence of primary lineages, each with distinct geographic structures, while dispersal events in the middle Pliocene and Pleistocene played a crucial role in shaping the diversity and distribution of existing species in Central and South America. In tandem, Hill et al. conducted research to understand the inheritance and expression of the easy-destemming trait with the aim of developing green chili pepper varieties suitable for mechanical harvesting. According to the data collected by studying populations derived from the landrace UCD-14 (male parent), the genetic basis of the destemming trait is quantitative with one major QTL on chromosome 10 and eight minor QTLs whose effects depend on the population and/or environment. The authors noted lignification occurring at the peduncle junction during fruit development in destemmed genotypes. The presence and activation of a peduncle/fruit abscission zone, controlled by genes located under several QTLs, appear to be responsible for the easy-destemming trait.

The breeding landscape expands further with Escalante et al.’s pioneering work on near-isogenic lines (NILs) of bell pepper, elucidating responses to viral infections. Escalante et al. released a data report describing two near-isogenic lines (NILs) of bell pepper cv. Marengo which were generated through backcross breeding. One of the NILs was infected with bell pepper endornavirus (BPEV) and the other remained BPEV-free. The authors released to the public the assembled transcriptomes and gene expression profiles of the two NILs, before and after infection with the acute virus pepper mild mottle virus (PMMoV). The data generated in this study opens avenues to comprehend interactions between persistent and acute viruses in plants, crucial for ensuring crop health and resilience.

Meanwhile, Martínez et al.’s exhaustive taxonomic study of *Physalis* species in Mexico serves as a cornerstone for conservation efforts. For each of the 61 recognized species, the authors have provided a wide range of information including morphological description, an artificial key (i.e., a set of morphological features) to determine species, data on geographical distribution and distribution maps, habitat, diagnostic characters, phenology, uses, photographs and finally the conservation status assessed by the International Union for Conservation of Nature (IUCN, 2023). This study serves as a vital resource for biodiversity conservation strategies in the region.

Transitioning to broader Solanaceae implications, Gebhardt **’**s integration of data from tomato and potato experiments sheds light on the genetic underpinnings of disease resistance and key agronomic traits. Gebhardt combined data from various experiments that utilized diverse mapping populations of tomato and potato, as well as different molecular markers. Data gathering revealed the co-localization of qualitative and quantitative loci associated with resistance to various pathogens, referred to as resistance hotspots. These findings suggest a common molecular basis for the response to biotic stress in both species. Further evidence indicates the existence of over 250 QTLs for tomato fruit and potato tuber sugar content, yield, and maturity. There are many genes underlying these QTLs that have pleiotropic effects on yield or resistance to pathogens or *vice versa*. Finally, the author points out that although QTL mapping has represented and still represents a useful tool for discovering genomic regions associated with traits of interest, genomic selection approaches will certainly prevail in the future.

Finally, Gagnon et al. **’**s comparative analysis unveils the ecological divergence of underground organs within the Solanum genus, shedding light on their adaptability to distinct environmental niches. The collected data demonstrated the environmental divergence of the two types of underground organs, with rhizomes and underground storage organs (USO) each occupying distinct disturbance and temperature regimes. This implies that geophytic organs are successful in different environments. The analyzed dataset, covering a wide variety of habitats worldwide, represents a significant step towards further phylogenetic and evolutionary studies based on macroecological scale.

In summary, the current Research Topic provides an overview of collective efforts that underscore an integrative approach to biodiversity conservation, climate resilience, and breeding innovations within the Solanaceae family. By intertwining evolutionary patterns, genetic insights, and ecological adaptations, these studies pave the way for more sustainable and resilient agricultural practices in the face of a changing climate and evolving agricultural needs.
